# Supplementary material for: Global Spread and Molecular Characterization of CTX-M-Producing Salmonella Typhimurium Isolates
Source: Antibiotics (Basel). 2021 Nov 19;10(11):1417. doi: 10.3390/antibiotics10111417 (PMC8614702; doi:10.3390/antibiotics10111417)
Supplement: Supplementary file 1 [file antibiotics-10-01417-s001.zip › antibiotics-1433786-supplementary.pdf]

Table S1. The detection rate of 154 virulence genes in 330 *bla*$_{CTX-M}$-positive *S.* Typhimurium isolates.

Virulence Gene	Rate	Virulence Gene	Rate	Virulence Gene	Rate	Virulence Gene	Rate
csgA	100.0%	*sscA*	100.0%	*sifB*	99.4%	*iucD*	3.0%
csgB	100.0%	*sscB*	100.0%	*sipB*	99.4%	*iutA*	3.0%
csgC	100.0%	*sseA*	100.0%	*sopA*	99.4%	*iroB*	1.8%
csgF	100.0%	*sseF*	100.0%	*sopE2*	99.4%	*iroC*	1.8%
csgG	100.0%	*sseG*	100.0%	*sptP*	99.4%	*iroD*	1.8%
fepG	100.0%	*steA*	100.0%	*csgD*	99.1%	*iroE*	1.8%
fimC	100.0%	*csgE*	99.7%	*lpfA*	99.1%	*iroN*	1.8%
fimD	100.0%	*entA*	99.7%	*lpfD*	99.1%	*cdtB*	0.6%
invA	100.0%	*fimF*	99.7%	*sinH*	99.1%	*faeD*	0.6%
invB	100.0%	*invI*	99.7%	*ssaD*	99.1%	*faeE*	0.6%
invC	100.0%	*invJ*	99.7%	*sseJ*	99.1%	*sspH1*	0.6%
invE	100.0%	*misL*	99.7%	*steC*	99.1%	*ybtP*	0.6%
invF	100.0%	*orgC*	99.7%	*ssaV*	98.8%	*ybtQ*	0.6%
invG	100.0%	*pipB*	99.7%	*sseC*	98.8%	*ybtS*	0.6%
invH	100.0%	*prgH*	99.7%	*ratB*	98.5%	*ybtX*	0.6%
mgtB	100.0%	*sifA*	99.7%	*slrP*	98.5%	*entE*	0.3%
mgtC	100.0%	*sipC*	99.7%	*entB*	98.2%	*faeC*	0.3%
ompA	100.0%	*sipD*	99.7%	*sodCI*	98.2%	*fyuA*	0.3%
orgA	100.0%	*sopB*	99.7%	*sseL*	98.2%	*irp1*	0.3%
orgB	100.0%	*sopD*	99.7%	*sopD2*	97.9%	*irp2*	0.3%
prgI	100.0%	*spaO*	99.7%	*mig-14*	97.6%	*senB*	0.3%
prgJ	100.0%	*spaR*	99.7%	*sseK2*	96.7%	*tviB*	0.3%
prgK	100.0%	*ssaC*	99.7%	*sseK1*	96.1%	*tviC*	0.3%
sicA	100.0%	*ssaM*	99.7%	*gogB*	91.8%	*tviD*	0.3%
sicP	100.0%	*ssaN*	99.7%	*pipB2*	89.4%	*tviE*	0.3%
sipA	100.0%	*ssaP*	99.7%	*sspH2*	83.0%	*vexA*	0.3%
spaP	100.0%	*ssaQ*	99.7%	*sseI*	79.7%	*vexB*	0.3%
spaQ	100.0%	*ssaR*	99.7%	*grvA*	31.8%	*vexC*	0.3%
spaS	100.0%	*ssaT*	99.7%	*spvB*	27.0%	*vexD*	0.3%
spiC	100.0%	*sseB*	99.7%	*spvC*	27.0%	*vexE*	0.3%
ssaE	100.0%	*sseD*	99.7%	*spvR*	27.0%	*ybtA*	0.3%
ssaG	100.0%	*sseE*	99.7%	*pefA*	26.7%	*ybtE*	0.3%
ssaH	100.0%	*steB*	99.7%	*pefB*	26.7%	*ybtT*	0.3%
ssaI	100.0%	*avrA*	99.4%	*pefC*	26.7%	*ybtU*	0.3%
ssaJ	100.0%	*fepC*	99.4%	*pefD*	26.7%		
ssaK	100.0%	*fimH*	99.4%	*rck*	26.4%		
ssaL	100.0%	*fimI*	99.4%	*shdA*	9.1%		
ssaO	100.0%	*lpfB*	99.4%	*iucA*	3.0%		
ssaS	100.0%	*lpfC*	99.4%	*iucB*	3.0%		
ssaU	100.0%	*lpfE*	99.4%	*iucC*	3.0%		

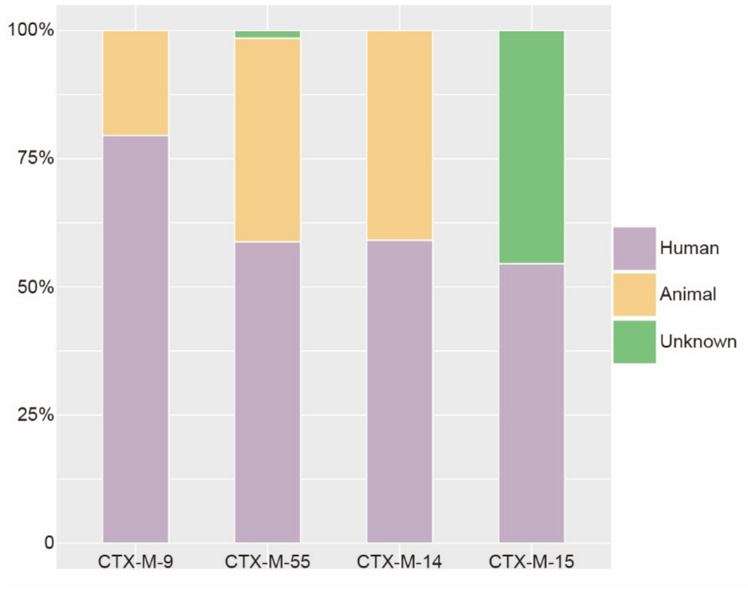

Figure S1. The analysis of hosts carrying the most common CTX-M allelic variants.
